# Supplementary figures and images for: PolyI:C Maternal Immune Activation on E9.5 Causes the Deregulation of Microglia and the Complement System in Mice, Leading to Decreased Synaptic Spine Density
Source: Int J Mol Sci. 2024 May 17;25(10):5480. doi: 10.3390/ijms25105480 (PMC11121703; doi:10.3390/ijms25105480)

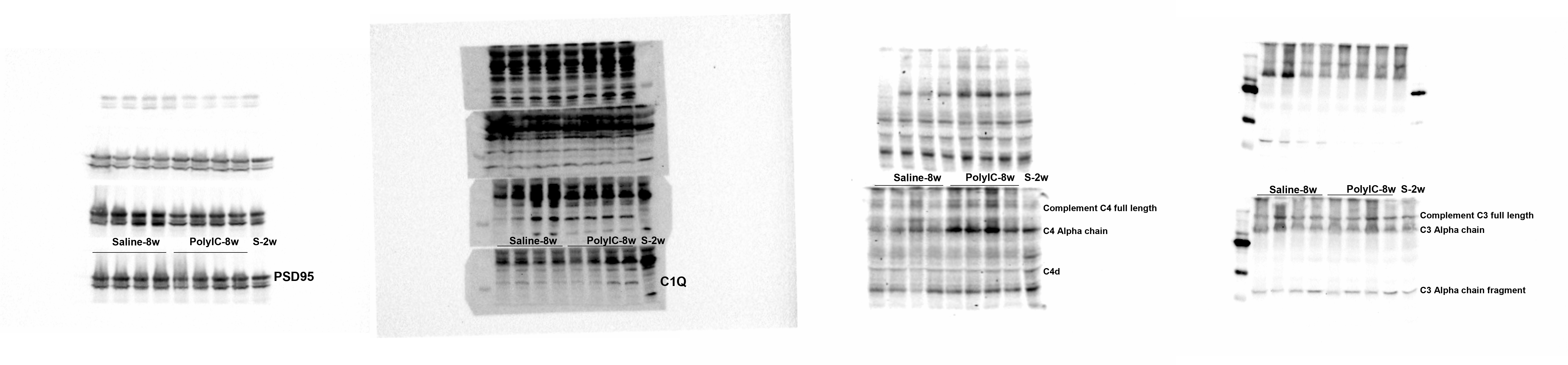

Supplement: Supplementary file 1 [file ijms-25-05480-s001.zip › 12w.tif]

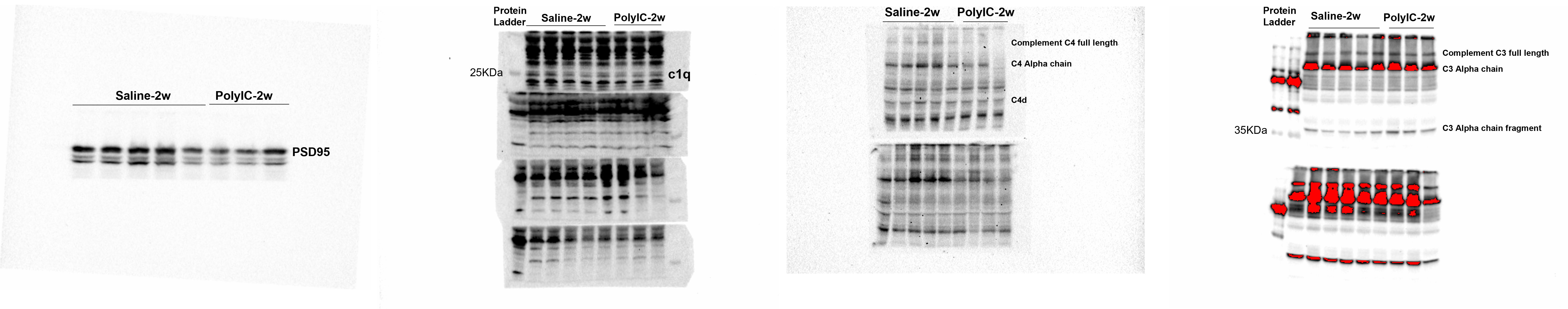

Supplement: Supplementary file 1 [file ijms-25-05480-s001.zip › 2w.tif]

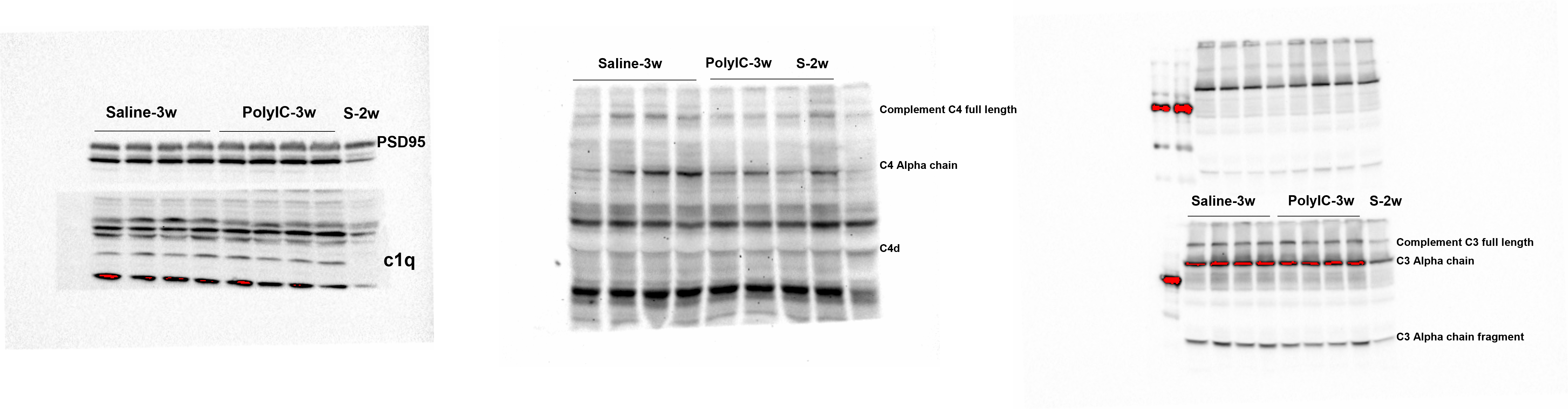

Supplement: Supplementary file 1 [file ijms-25-05480-s001.zip › 3w.tif]

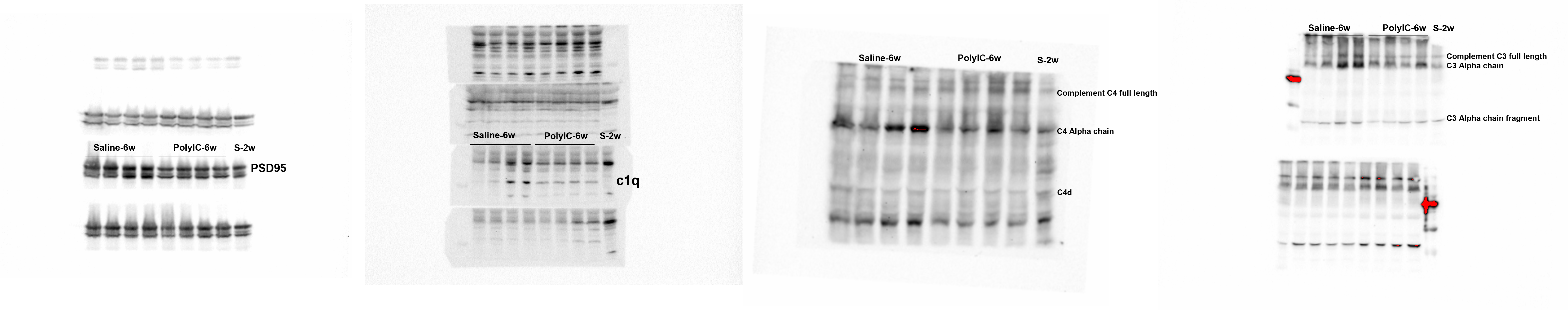

Supplement: Supplementary file 1 [file ijms-25-05480-s001.zip › 6w.tif]

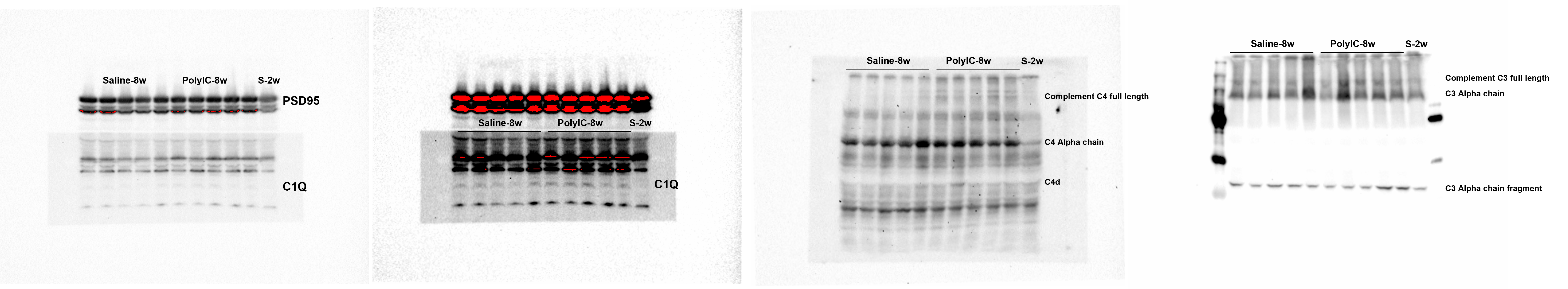

Supplement: Supplementary file 1 [file ijms-25-05480-s001.zip › 8w.tif]
